# Rhino-Orbito-Cerebral Mucormycosis: Two Cases with Amaurosis as Presentation, Medical Surgical Management and Follow-Up

**DOI:** 10.1155/2019/4215989

**Published:** 2019-12-05

**Authors:** Carmen Navarro-Perea, Ignacio Cañas-Zamarra, Enrique Mencía-Gutiérrez, Enrique Revilla-Sánchez, María-Dolores Lago-Llinás, Silvia Pérez-Trigo, Álvaro Bengoa-González

**Affiliations:** ^1^Ophthalmology Department, 12 de Octubre Hospital, Complutense University, 28041 Madrid, Spain; ^2^Pathology Department, 12 de Octubre Hospital, Complutense University, 28041 Madrid, Spain

## Abstract

*Purpose:* Mucormycosis is an infection caused by fungi to the class Zygomycetes that usually appears in immunosuppressed patients. Diagnostic confirmation is often delayed, with fatal prognosis in cases in which treatment is not rapidly established. *Case report:* We present two clinical cases of rhino-orbito-cerebral mucormycosis with an atypical presentation form, consisting of a unilateral complete sudden vision loss. Intravenous treatment with liposomal amphotericin B was started and total orbital exenteration surgery was performed. The removed surgical area was filled with gauze impregnated with liposomal amphotericin B and was left open for cures every 12 hours. Due to the good clinical evolution, a reconstruction of the orbital exenteration defect was performed in Case 1 with a temporal muscle flap and a skin island pedicled flap. In Case 2, reconstruction was not performed due to the poor evolution of the patient. *Discussion:* As it is a very aggressive surgery, the aesthetic and functional sequelae are very important. When the survival of the patient is achieved, we should offer reconstructive solutions that improve their quality of life. The reconstruction carried out using a flap of the temporal muscle can be made in a single act without requiring microvascular surgery.

## 1. Introduction

Mucormycosis is an opportunistic and severe infection caused by fungi of the order of Mucormycetes and class Mucorales that usually appears in immunosuppressed patients. The most common agents of mucormycosis are Rhizopus spp., Mucor spp. and Lichtheimia (formerly Absidia and Mycocladus) spp. Genera of other Mucorales, such as Rhizomucor, Sakseanea, Cunninghamell, and Apophysomyces, are less common [1, 2]. It is infrequent, although it is difficult to know its exact incidence because it is not a notifiable disease. However, the current trend reveals an increase in the incidence of fungal infections. Mucormycosis is the third most common fungal infection after those produced by *Candida* spp. and *Aspergillus* spp. [3]. The most commonly associated comorbidities include: diabetes mellitus, hematologic processes (hematologic malignancies, severe neutropenia, graft-versus-host disease, hematopoietic stem cell transplantation), solid organ transplant, injection drug use, malnutrition, treatment with corticosteroids, burns, severe traumatisms and renal failure [4]. Mucormycosis is classified in six categories: rhino-orbito-cerebral (ROC), pulmonary, cutaneous, gastrointestinal, disseminated and other unusual presentations as [5], with ROC being the presentation in 40% of the cases [6]. A new category has recently been described: isolated renal mucormycosis, but it is rare and has been described mainly in developing countries like India and China. It is rarer still to find this entity in immunocompetent young males without any risk factors [7].

Diagnosis of mucormycosis is based on the identification of the microorganisms in the pathological study with a microbiological culture (although it is usual to find no growth). The endoscopic evaluation of the sinuses with presence of necrosis, incisional biopsy and radiological image are very important for diagnosis. Diagnostic confirmation is often delayed, an important fact considering the fatal prognosis in the cases in which treatment is not rapidly established [3].

We present two clinical cases of ROC mucormycosis with an atypical presentation form, consisting of a unilateral sudden vision loss. In a review of the literature we have found only one case with a similar presentation [8].

## 2. Case 1

**Figure 1:**
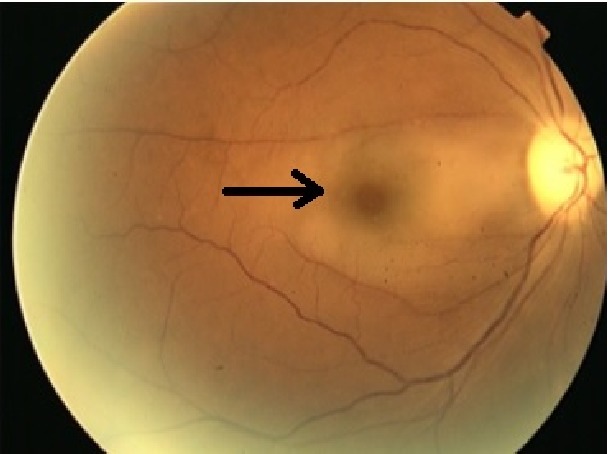
Case 1, fundus photography showed obstruction of the central artery of the retina (cherry red spot (arrow)) and posterior pole edema affecting right eye.

**Figure 2:**
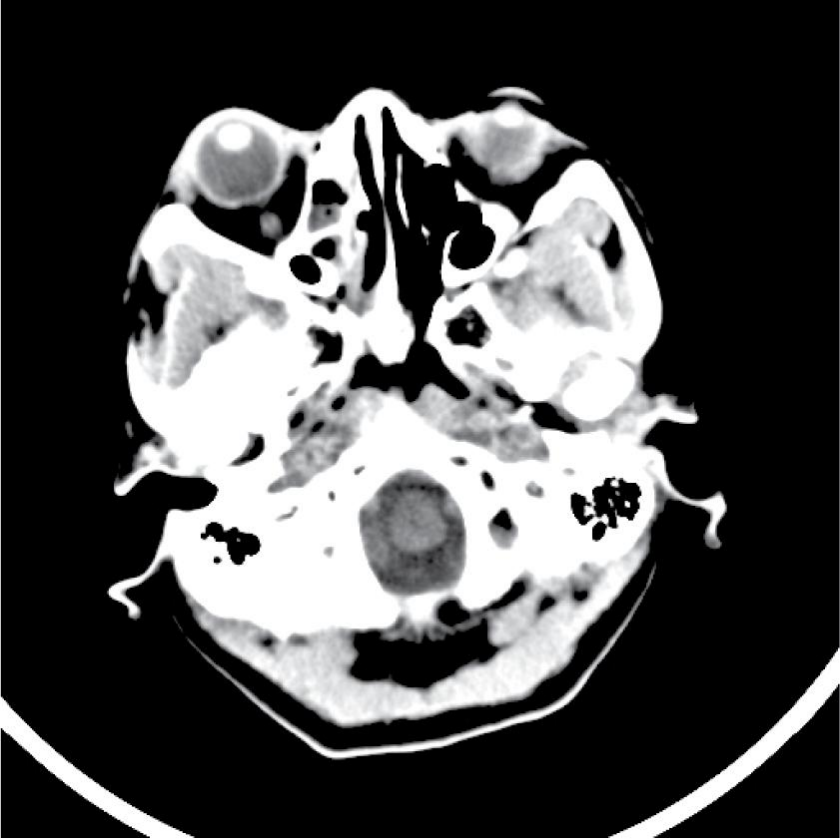
Case 1, computed tomography (CT) with partial occupation of right ethmoidal cells and right sphenoid sinus.

**Figure 3:**
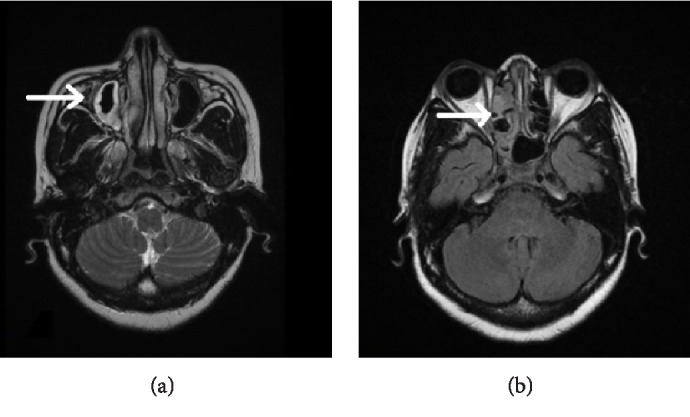
Case 1, magnetic resonance with right ethmoid and sphenoid occupation with increased compared to previous CT (Figure 2).

A 50-year-old female, with a renal transplantation the previous year due to chronic renal failure of unknown etiology, arrives at Emergency due to a sudden loss of vision in the right eye (RE) and a right hemicranial headache focused in the temporal area. In the ophthalmological assessment visual acuity of the RE was nonperception of light (NPL); in the fundus there was macular and posterior pole edema with cherry red spot, and reduction of the vascular caliber with sharp arteries and segmentation of blood flow (Figure 1). In the analytical study we observed a slightly increased erythrocyte sedimentation rate and normal C-reactive protein. A computed tomography (CT) was requested in which a partial occupation of right ethmoidal cells and right sphenoid sinus was observed (Figure 2). Nasofibroscopy was normal despite the CT findings. The initial diagnosis was a central retinal artery obstruction. The patient was reassessed 24 hours later due to the lack of any clinical improvement. A magnetic resonance (MR) was performed, which showed right ethmoid and sphenoid occupation with increased density compared to the previous CT, but with no signs of invasion (Figures 3(a) and 3(b)). A new nasofibroscopy demonstrated necrosis of the middle turbinates with purulent discharge, necrosis and sloughing in the uncinate process and in the right middle meatus and on the inferior turbinates. Samples were taken for bacterial and fungal culture and for direct examination and Gram stain, as well as for pathological study. In the direct examination structures of a nonseptate filamentous fungus were observed, compatible with mucormycosis.

**Figure 4:**
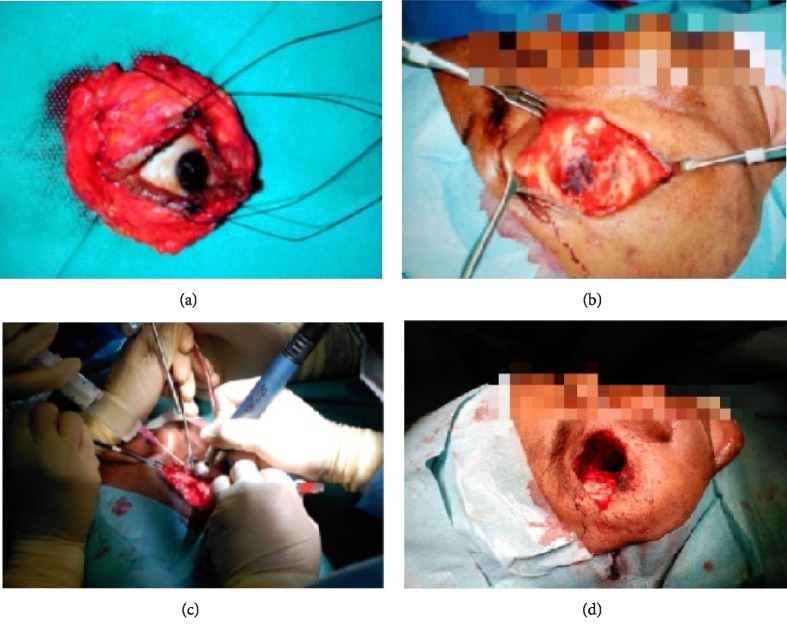
Case 1 (a) urgent surgical intervention with total exenteration of the right orbit (b) (c) (d) orbital cavity exenteration what the surgical bed shows.

Given these findings, intravenous (iv) treatment with liposomal amphotericin B was started at 5 mg/kg dose, anidulafungin and meropenem 1 g/8 h. Urgent surgery was performed for exeresis of the affected tissues; it consisted of: medium meatotomy, uncinectomy, ethmoidal bulla resection, anterior and posterior sphenoid sinus with necrotic and friable mucosa, resection of papyracea lamina mucosa, middle turbinate resection, transnasal sphenoidectomy and inferior turbinate resection. All the tissues were sent for microbiological and pathological study. Suspecting ROC mucormycosis, total orbital exenteration was performed, including the skin of the eyelids, in which necrosis of the medial wall and orbital floor was found, so a broad debridement was performed (Figures 4(a)–4(d)).

**Figure 5:**
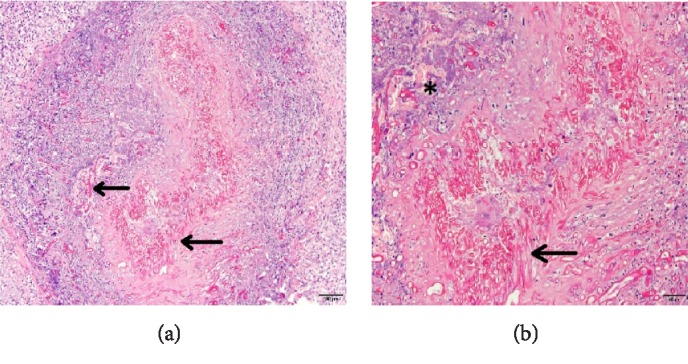
Case 1 pathological study (a) and (b), pathological examination showed abundant wide mycelia (arrow), nonseptate, some of them partitioned, that were arranged angiocentrically, destroying and invading the vascular walls. (a) Hematoxylin&Eosin stain, original magnification × 10, 100 *μ*m scale lower right. (b) Hematoxylin&Eosin stain, original magnification × 20, 50 *μ*m scale lower right. There were fungal microabscesses with necrotic center in periphery (b) (asterisk).

**Figure 6:**
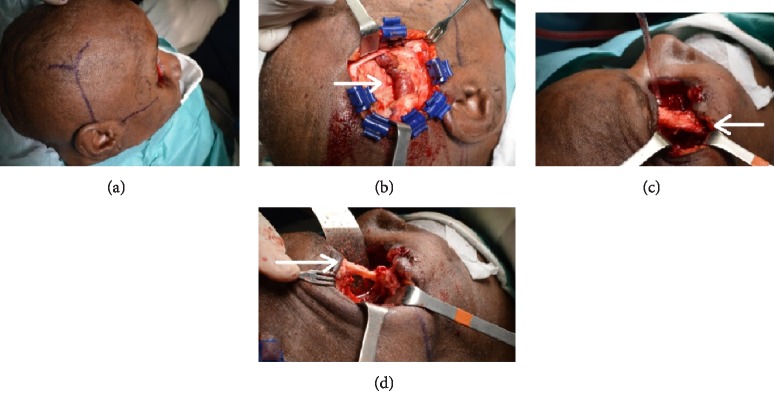
Case 1 (a) reconstructive surgery, (a) temporal cutaneous marking of the right orbital exenteration with a temporal muscle flap (b) (arrow) through a modified lateral orbitotomy (bone window in the lateral wall maintaining the external part of the frontal malar apophysis (c) and (d) (arrow). Initial to mid-stage surgery.

**Figure 7:**
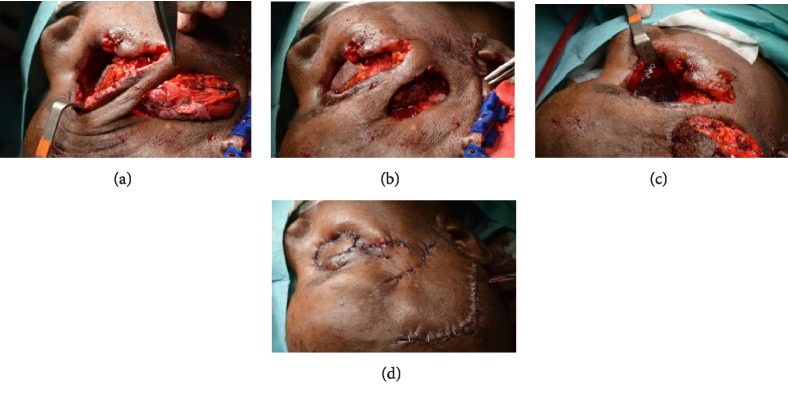
Case 1, (a)–(d) reconstructive surgery of the right orbital exenteration with a pedicle muscle temporal through a modified lateral orbitotomy (bone window in the lateral wall maintaining the external part of the frontal malar apophysis) and a skin pedicle flap. Final stage of surgery.

**Figure 8:**
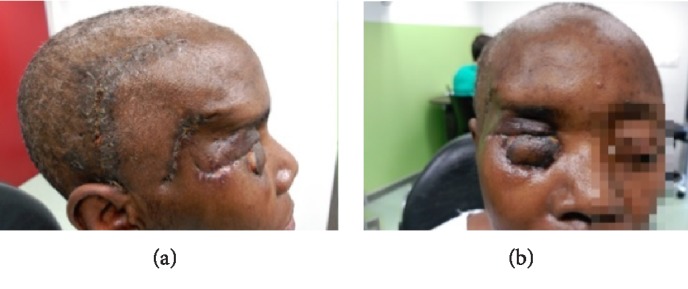
Case 1 (a) and (b) clinical aspect of the patient 2-weeks after reconstruction surgery.

**Figure 9:**
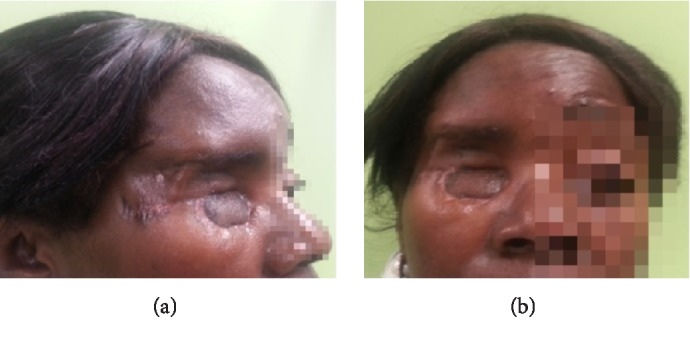
Case 1 (a) and (b) clinical aspect of the patient 6-months after reconstructive surgery.

The removed surgical area was filled with gauze impregnated with liposomal amphotericin B and the wound was left open for cures (replacement of gauzes) and exploration every 12 hours. The Nephrology service modified the treatment in order to reverse the immunosuppression to which she was subjected due to the kidney transplant: the administration of mycophenolate was suspended and the dose of tacrolimus was reduced until reaching an objective level of 5 mg/ml. On the fourth postoperative day, necrotic tissue in the roof of the orbit and upper area of choana was debrided. One week after the surgery, due to the good evolution, the cures were scheduled every 24 hours. Pathological examination showed abundant wide mycelia (arrow), nonseptate, some of them partitioned, that were arranged angiocentrically, destroying and invading the vascular walls. There were fungal microabscesses with necrotic center in periphery (asterisk) (Figures 5(a) and 5(b)). The culture confirmed *Rhizopus oryzae*. At the first postoperative month, thanks to the recovery of immunosuppression of the patient and the good clinical evolution, the reconstruction of the orbital exenteration defect was performed with a temporal muscle flap and a skin island pedicled flap, and a titanium mesh implant in medial and lower region (Figures 6(a)–6(d) and 7(a)–7(d)). Postoperative images two weeks after the reconstruction (Figures 8(a) and 8(b)) and after 6 months (Figures 9(a) and 9(b)) are shown.

Two years later, there are still no signs of infection or necrosis, and the reconstructed area looks alright.

## 3. Case 2

A 54-year-old female with diabetes mellitus type 2, arterial hypertension and chronic pancreatitis presented at Emergency due to unilateral loss of vision of the RE. In the ophthalmological examination, visual acuity was NPL. Six hours later there were palpebral ptosis and a complete oculomotor paralysis of the RE. Those signs had not been present in the first examination.

**Figure 10:**
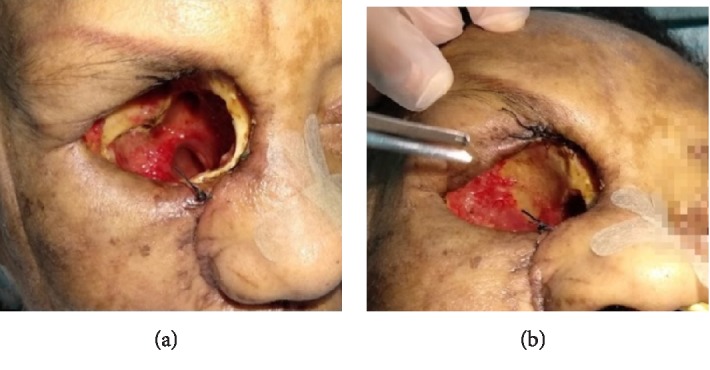
Case 2 (a) and (b) 7-days after postsurgery of right orbital total exenteration.

**Figure 11:**
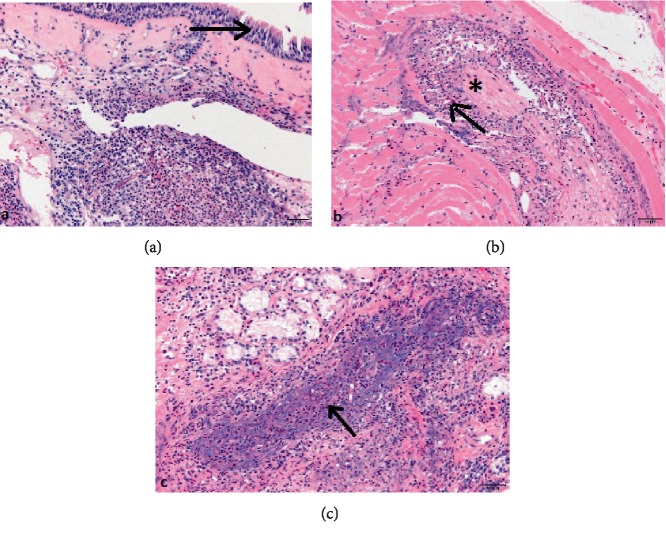
Case 2 (a) pathological study showed a respiratory epithelium (pseudostratified ciliated) (arrow). In the underlying chorionnonseptate hyphae associated with an acute neutrophilic infiltrate were identified, Hematoxilin&Eosin, original magnification × 20 (50 *μ*m scale lower right). In (b) associated nonseptate hyphae (arrow) and a neutrophilic inflammatory infiltrate appear around a nerve (asterisk) Hematoxylin&Eosin, original magnification × 20 (50 *μ*m scale lower right) and in (c) there are also nonseptate hyphae (arrow), cellular debris and a neutrophilic infiltrate that destroys the wall of a blood vessel, Hematoxylin&Eosin stain, original magnification × 20 (50 *μ*m scale lower right).

An orbital CT showed right orbital cellulitis, with limited frontoethmoidal occupation and optic ischemic neuritis (apex orbitary syndrome (AOS)). Antibiotic treatment was started with no response. 24 hours later, endoscopic nasosinusal surgery was performed and signs of nasal mycosis were found. Given the poorly controlled diabetes mellitus, a fungal orbital cellulitis was suspected, and an MR was performed 48 hours after the beginning of the symptoms; there was a strong suspicion of invasive fungal sinusitis with orbital involvement. Samples of hard palate were taken and sent to microbiology and histopathology study, reaching the diagnosis of mucormycosis by direct examination with the presence of wide nonseptate mycelia. Immediate intravenous treatment was established, following the same pattern as in the previous case, and total orbital exenteration with maxillectomy was performed (Figures 10(a) and 10(b)), keeping part of the orbital floor, complete ethmoidectomy and sphenoidectomy with opening of lateral recess, frontal sinus opening by external approach and resection of hard hemipaladar. The removed surgical area was washed with liposomal amphotericin B and filled with gauze impregnated with it, and the surgical wound was left open for cures and exploration every 12 hours. One week after surgery, due to good evolution, cures were scheduled every 24 hours. Pathological study showed a respiratory epithelium (pseudostratified ciliated) (arrow). In the underlying chorion, nonseptate hyphae associated with an acute neutrophilic infiltrate were identified (Figure 11(a)). In (Figure 11(b)) associated nonseptate hyphae (arrow) and a neutrophilic inflammatory infiltrate appear around a nerve (asterisk). And in (Figure 11(c)) there are also nonseptate hyphae (arrow), cellular debris and a neutrophilic infiltrate that destroys the wall of a blood vessel. Microbiological culture showed *Rhizopus oryzae* and sequencing 18S ARNr: PCR confirmed it.

During follow-up, the patient was diagnosed with pulmonary epidermoid carcinoma in the left upper lobe with mediastinal invasion. For this reason, the reconstruction of the orbital defect was not performed.

## 4. Discussion

Rhizopusis the most common genera and is predominantly observed in patients with ROC mucormycosis [9]. It is a species of ubiquitous and saprophytic fungus that may be found in decomposing organic matter and forms spores that are easily inhaled by humans. In immunosuppressed hosts, the fungus can easily invade blood vessels, which causes thrombosis, hemorrhage and necrosis, which are the characteristic pathological findings found in this type of infection [10]. Mucorales generally affects immunosuppressed people, with diabetes mellitus being the most important risk factor [11]. The reason is that diabetic patients show a decrease in the phagocytic function of neutrophils, myeloperoxidase activity and adherence of neutrophils to the endothelial wall. In addition, the states of acidosis and hyperglycemia suppose a perfect environment for the growth of fungi [12].

Although infection by Mucorales can appear in different locations, the most frequent is the ROC presentation (40%). In ROC mucormycosis, the infection progressively spreads from the nasal mucosa to the facial sinuses, palate, orbits and brain. The nasal and sinus involvement is usually asymptomatic or paucisymptomatic, so it can easily go unnoticed. Patients usually consult when the infection extends, reaching the orbital region. The most frequent symptoms and signs are palpebral edema, ptosis, decreased visual acuity and headache [3].

Early diagnosis and the combination of surgical and pharmacological treatment established in the first 5 days improve prognosis, showing a reduction in the 3-month mortality rate of 

48.6% [12]. Patients treated in the first 5 days have a survival rate of 76–81%, while in those treated after the first 12 days the survival rate is reduced to 36–42% [13, 14].

We report two cases of ROC mucormycosis that started with sudden unilateral amaurosis. In the literature we found that the appearance of this symptom is histologically related to the occlusion of the central artery of the retina, necrosis of the ophthalmic artery, with infarction and necrosis of the optic nerve or with direct infection of the optic nerve by Rhizopus [15].

In Case 1, an obstruction of the central artery of the retina was diagnosed, with the nasofibroscopy with sampling providing the diagnostic confirmation of mucormycosis. Case 2 presented with unilateral amaurosis and, several hours later, with a compatible clinical image with OAS. OAS is a rare syndrome that presents with ophthalmoplegia, vision loss and retro-orbital pain [16]. In the literature, it is widely shown that OAS can be the first symptom of mucormycosis [3], but in these cases the first symptom was amaurosis.

Success in the treatment of mucormycosis depends on the following factors: early diagnosis, urgent debridement of the necrotic tissues and resection of infected tissues, parenteral medical treatment and the correction of the predisposing factors in cases where it is possible, with the reversal of immunosuppression being one of the most important factors [17]. Surgery with exeresis of all infected tissues reduces mortality by 49% according to the study published by Tedder et al. [18]. This percentage rises to 71% when the treatment with liposomal amphotericin B, the gold standard treatment in mucormycosis, is added [3, 19]. Mucormycosis produces vascular obstructions, which can hinder the penetration of iv amphotericin into tissues invaded by the fungus. For this reason, the surgical wound is left exposed, in order to be able to irrigate it with liposomal amphotericin B, in combination with the systemic treatment [13, 18]. This also allows the daily assessment of the wound and the resection of any new necrotic tissue that may appear. The therapeutic approach taken, together with the improvement of the patient's immune status, achieved complete healing in both cases, so multidisciplinary management is essential for successfully treating infection. In a period of approximately one month, the complete resolution of the process was achieved, allowing subsequent reconstruction.

As it is a very aggressive surgery, the aesthetic and functional sequelae are very important for the patient. In the cases in which the survival of the patient is achieved, such as those we have presented previously, it is fundamental to offer reconstructive solutions that improve their quality of life once the infection is resolved. The orbital reconstruction carried out using a flap of the temporal muscle, allows the realization of the technique in a single surgical act, without the need for microvascular surgery. In addition, this flap presents adequate vascularization and the healing time is shorter than in other techniques [20, 21]. This procedure is completed with a skin island pedicled flap, avoiding the need to take skin grafts from other locations and reducing the risk of necrosis; with the implant of a titanium mesh in the medial and lower regions, the aesthetic result is very satisfactory for the patient, as in Case 1. We did not find any description of the island pedicled skin flap for reconstruction in the literature.

## 5. Conclusions

In conclusion, mucormycosis is an uncommon but highly lethal infection, and it is very important to keep this diagnosis in mind, in order to begin treatment early and increase the chances for survival. In addition, a multidisciplinary approach is essential to achieve these objectives. We consider the communication of these clinical cases essential in order to continue improving the therapeutic approach to this infection, to reduce the mortality rate and, in those patients in which survival is achieved, to improve their quality of life.

## Data Availability

The data that support the findings of this study are available from the corresponding author, CN-P, upon reasonable request.
